# Investigation of acoustic waves behavior of an underground tunnel in a multilayer soil

**DOI:** 10.1038/s41598-022-16991-w

**Published:** 2022-08-04

**Authors:** A. Roohezamin, R. Kalatehjari, M. Hajihassani, M. Kharghani, D. Dias

**Affiliations:** 1 Department of Civil Engineering, K. N. Toosi University of Technology, Tehran, Iran; 2 Built Environment Engineering Department, School of Future Environments, Auckland University of Technology, Auckland, 1010 New Zealand; 3 Department of Mining, Faculty of Engineering, Urmia University, Urmia, 5756151818 Iran; 4 Department of Mining, Faculty of Engineering, Islamic Azad University, Science and Research Branch of Tehran, Tehran, Iran; 5 Department of Civil Engineering, Université Grenoble Alpes, Laboratoire 3SR, 38610 Gières, France

**Keywords:** Engineering, Civil engineering, Geophysics

## Abstract

Understanding the acoustic behavior of buried tunnels is valuable for locating them and monitoring their structure health. This research focuses on the acoustic behavior of buried tunnels in multilayer soil structures. The reflected and transmitted acoustic wave pressure variations are investigated exclusively for a multilayer soil buried tunnel. The tunnel system's 3D finite element model is presented, which contains the tunnel lining, surrounding soil, and the air inside the tunnel and at the ground surface. A free air explosion is used as the acoustic wave source. The reflected and transmitted waves' pressure values are measured to evaluate the effects of mechanical characteristics of soil layers, tunnel buried depths, and lining concrete types on the acoustic wave behavior of the tunnel. In addition, a utility line is introduced to the system in different positions related to the main tunnel to investigate its effect on the main tunnel’s acoustic wave behavior. The results indicate that in a multilayer soil structure, the relative position of the soil layers and the tunnel (whether the main tunnel or the utility line) significantly impacts the acoustic pressure value, particularly the transmitted wave pressure. When changing the tunnel buried depth and the lining concrete type, multiple pressure peaks are observed in reflected acoustic wave pressure–time history exclusive to a tunnel surrounded by a multilayer soil structure. The findings can be used to precisely interpret the recorded signals for structural health monitoring and locating underground structures, especially in a media with multilayer soil structures.

## Introduction

In recent decades, the demand for underground facilities such as water distribution and subsurface transportation systems has increased due to urban areas and urbanization developments. Finding the precise location and conducting health monitoring of such subsurface structures are usually complex and costly due to accessibility difficulties after construction. Therefore, researchers are investigating alternative techniques, such as acoustic wave-based methods. Analyzing the behavior of the acoustic wave generated by an aboveground source and radiated towards the buried structures is an essential part of the acoustic wave methods because in such techniques, the recorded signals are affected by the propagation medium and, in consequence, are misinterpreted^1^.

Analyzing the transmitted and reflected acoustic waves when interacting with different components of a buried structure provides a comprehensive knowledge of the acoustic wave behavior, which results in a more realistic interpretation of the recorded signals for acoustic wave-based methods. The effects of soil properties on acoustic wave behavior have been studied in the literature; however, limited studies considered the surrounding soil of the buried tunnel as a multilayer medium. Single-layer soil structure is hardly observed in the real world when dealing with buried structures. Therefore, considering the layered soil structure is more realistic and provides more comprehensive knowledge about the acoustic wave behavior for locating or health monitoring an underground structure.

Studying the interaction between the soil layers and its effects on the radiated acoustic wave behavior is necessary when considering the layered structure of the soil surrounding a buried structure. Different geological properties of the soil layers affect how the acoustic waves reflect and transmit, particularly at the soil layers' interfaces. Consequently, the recorded acoustic wave reflected from the buried structure is influenced by the layered structure of the surrounding soil. Additional complexity is introduced to interpreting the reflected and transmitted acoustic waves if the underground structure is located between the soil layers.

Several studies studied the acoustic wave and its applications in various fields, particularly buried structures. Varadan and Vasundara^2^ reviewed the longitudinal and shear waves scattering from an elliptic cross-section cylinder by applying the scattering-matrix method and found it suitable to calculate the elastic wavefield spread from an obstacle. By utilizing the wave functions expansion approach, Chen^3^ analyzed the dynamic stresses of a cylindrical tunnel liner under a horizontal shear wave to calculate the dynamic stress concentrations for various parameters. Hua et al.^4^ investigated the dynamic stresses of underground circular lining tunnels under plane P waves. They represented a wave equation series solution for the dynamic response of the tunnel by using the Fourier–Bessel series expansion method. Romero et al.^5^ investigated the acoustic wave propagation from underground structures and studied the noise and vibrations reflected from a tunnel using a 2.5D boundary element-finite element (BEM–FEM). They demonstrated that the soil-structure interaction impacted the sound pressure field above the ground surface. Liao et al.^6^ analyzed the acoustic waves scattering by a buried tunnel applying the transition matrix (T-matrix) theory considering an oblique propagation of the incident wave to illustrate the scattered and refracted wave fields in series forms.

The acoustic waves induced by subsurface cavities—treated as buried structures—were studied by some researchers. Lancioni et al.^7^ numerically analyzed the effects of cavities inside homogenous soils and the seismic shear waves caused by them. The same situation was considered by Lin et al.^8^, who proposed a new method to quantify the wave scattering and diffraction from underground cavities in a layered half-space. Thambiratnam and Lee^9^ proposed a simplified approach to deal with the shear wave scattering by cavities with arbitrary shapes. Chen^10^ studied the scattering of S.H. waves by U-shaped cavities considered equivalent to underground tunnels in a half-space and found they remarkably affect the wave propagation and influence the incident wave number, depth, and angle.

The health monitoring of underground structures is another application of acoustic wave-based methods that have received attention from researchers. Zhu et al.^11^ applied acoustic signals to monitor probable damage and blockage of the underground siphons. Liu et al.^12^ found another application for the acoustic waves in pile health monitoring by locating karst cavities in the vicinity of the piles. Knag et al.^13^ examined buried underground tunnels' location, scale, and damage mechanisms by simultaneously monitoring the reflected acoustic emission and the surrounding soil damage progress. Hu et al.^14^ and Cheng et al.^15^ carried out the same procedure for monitoring damage development in surrounding soil during tunnel excavation by TBM. Lyapin et al.^16^ discussed acoustic wave applications in monitoring underground structures of arbitrary shapes in layered soil media by developing an analytical solution. By conducting a series of field vibration tests in buried tunnels, Zhou et al.^17^ investigated the acoustic properties of underground structures. They checked the possibility of using acoustic waves to monitor the structure's health.

Locating underground structures is another topic involving the usage of acoustic waves. Several researchers applied the acoustic wave for the leakage detection and locating of pipelines. Jeong et al.^18^ tried to find utility lines like gas and sewer systems by applying the acoustic transmission method. Muggleton and Brennan^19^ proposed an instrument to detect the location of underground pipe systems by acoustic methods. Fuchs and Riehle^20^, Liang et al.^21^, and Liu et al.^22^ applied the acoustic wave methods in pipeline leakage detection. Butterfield et al.^23^ used an acoustic emission approach to quantify the leakage rate in water distribution pipes. Dong et al.^24^ experimentally investigated the effects of temperature on the acoustic emission source's accuracy, which leads to reducing errors in locating the structural damages caused by temperature. Finally, Jia et al.^25^ and Kim et al.^26^ found acoustic wave methods effective in underground structure monitoring, especially concrete structures.

Different characteristics of the elements interacting with the propagated waves in an underground structure system should be investigated to comprehensively understand the reflected and transmitted acoustic wave behavior. Several researchers, including Long et al.^27,28^, studied soil characteristics' effects on acoustic waves' behavior and dispersion in underground structures. Jones^29^ and Iodice et al.^30^ used in-situ soil measurement tests to quantify the soil effect on acoustic waves. Massah et al.^31^ used the boundary element method to study the impact of soil surrounding the tunnel on the acoustic wave. Massah and Torabipour^32^ investigated the effects of other parameters like the tunnel buried depth, lining concrete material, and lining thickness on the behavior of reflected and transmitted acoustic waves from a buried tunnel in a single-layer soil structure. Han et al.^33^ studied multilayer soil structures, where the soil-underground structure interaction was investigated considering the pore pressure effect and the dynamic characteristics of the site. In a similar study, Meng et al.^33^ simulated and investigated the propagation of shock waves across multilayer structures under an underwater explosion. Aristizábal-Tique et al.^34^ also analyzed a multilayer soil structure and its interaction with a buried slab by considering one-dimensional ray tracing and quantified the reflected and transmitted acoustic waves from the buried structure.

The present study investigates the behavior of reflected and transmitted acoustic waves in a 3D numerical model of an underground structure excavated in multilayer soil which has not been exclusively studied previously. The investigation includes the influences of the surrounding soil, soil-tunnel interaction zone, and soil layers on the acoustic wave behavior. A 3D model of a subway tunnel surrounded by a three-layer soil is modeled in the finite element ABAQUS commercial software under a free air explosion. The reflected acoustic wave from the tunnel and the wave transmitted into the tunnel are analyzed for various soil properties, tunnel buried depths, lining concrete models, and different positions of extra second underground structures. The finding of this study can be used for locating and structural health monitoring of underground tunnels with higher accuracy.

## Numerical modeling

### An introduction to the proposed model

**Figure 1:**
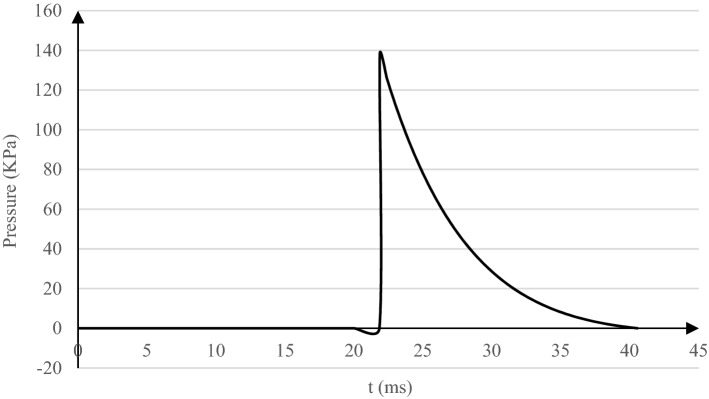
Overpressure time history of a 500 kg TNT explosion, 20 m above the ground surface.

The proposed model is simulated based on the Tabriz Urban Railway Line 2’s geometrical properties in Iran^35^. The inner diameter of this tunnel circular section is equal to 9.0 m with a 0.35 m-thickness lining. The acoustic wave radiates through the soil, and the underground tunnel is generated by a 500 kg TNT explosion located at 20 m above the soil surface in the model's center. Based on the curves and relations accessible in UFC^36^, such a free air explosion, producing a maximum overpressure of 137.89 kPa, is initiated at t = 0. After 21.88 ms, it reaches its peak overpressure value and diminishes to zero pressure at t = 40.55 ms. The explosion overpressure time history which is applied to the model as an “incident wave,” is illustrated in Fig. 1, where the negative part is neglected due to its insignificant effect on the final results.

**Figure 2:**
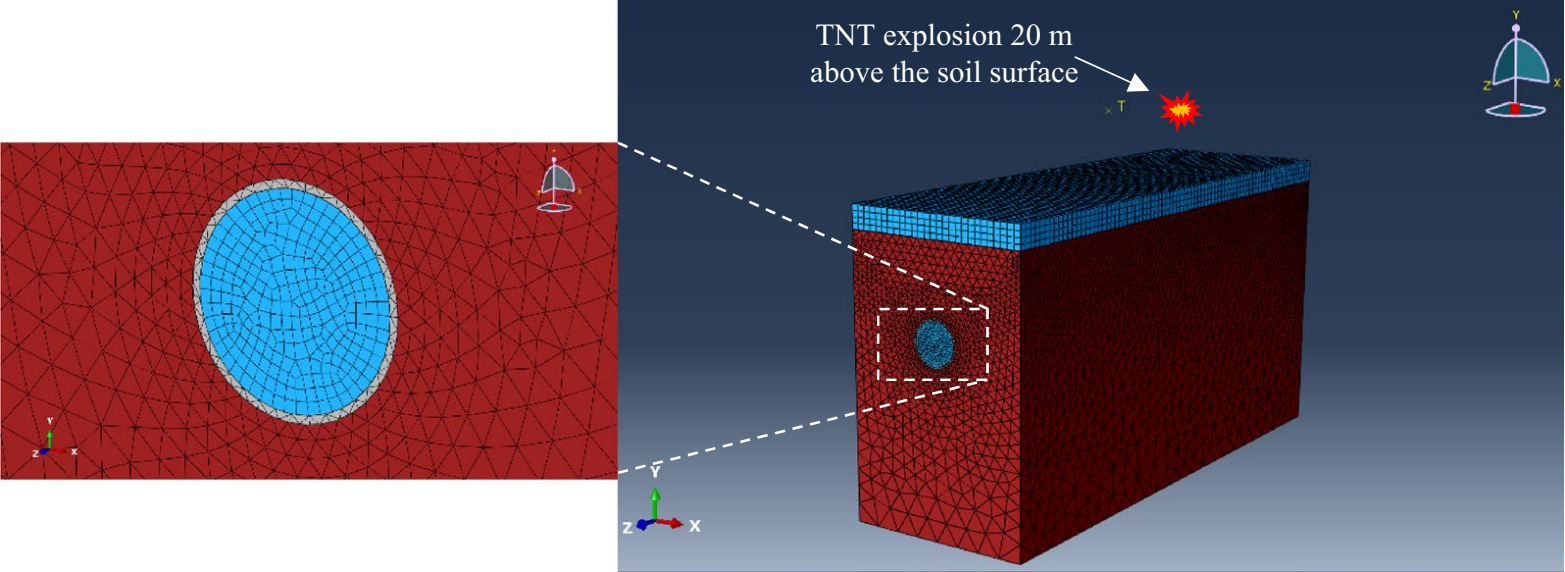
Schematic representation of the underground structure system where red represents the soil, grey represents the lining, and blue represents the air both inside the tunnel and at the surface.

The numerical model was created using the finite element ABAQUS numerical code, as shown in Fig. 2. The model includes the lining concrete, the soil surrounding the tunnel, the air inside the tunnel, and the air at the ground surface were considered under a TNT explosion 20 m above the ground.

As mentioned before, the model dimensions along the z and y*-*axis (see Fig. 2) are considered to be respectively equal to 120 and 60 m. The 3D numerical model is made of 14,400 SC6R wedge elements, 314,645 C3D4 tetrahedron elements, 53,531 and 8640 AC3D8R hexahedron elements. The ABAQUS/Explicit solver was used to solve the considered problem. However, using an explicit dynamic method arises some limitations, like applying the proper energy absorption mechanism at the far end boundaries.

**Figure 3:**
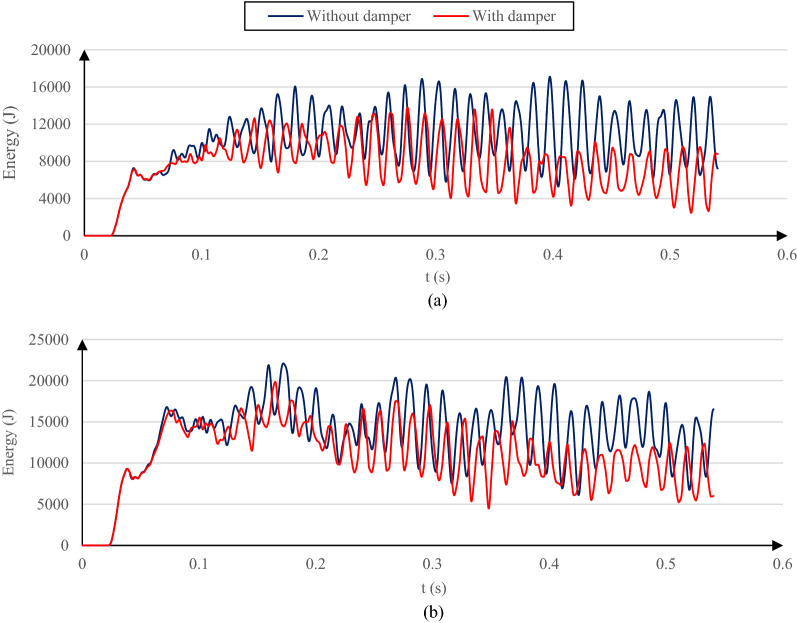
Model (**a**) kinematic and (**b**) strain energies, with and without the proposed damping mechanism.

To attenuate the energy propagated towards the model far ends, it is common to introduce viscous dampers in the three orthogonal directions at the model far end boundaries based on the approach proposed by Lysmer and Kuhlemeyer^37^. In ABAQUS, such a procedure is accomplished by assigning dashpot elements to all nodes of the solid parts at the far end boundaries. However, these specific elements are not supported by the explicit version of ABAQUS. To overcome this issue, two rigid planes acting as the ground are modeled with an appropriate distance from the far end boundaries (along the z-axis), and fasteners are applied between the model far ends nodes and the parallel rigid planes. Proper damping coefficients (deduced from^37^) were assigned to the fasteners; the desired damping mechanism is fulfilled. As depicted in Fig. 3, the model kinematic and strain energies are reduced by applying the proposed damping mechanism at the far end boundaries of a model with the mentioned geometrical properties and a single-layer soil.

The proposed model used three soil layers to investigate the acoustic wave behavior in an underground tunnel with multilayer soil. The geological properties of soil layers are sourced from the borehole BH-23 excavated for the S03 station of Tabriz Urban Railway Line 2^38–40^ and are represented in Table 1.

**Table 1:** Geological properties of soil layers.

Soil layers	Layer 1	Layer 2	Layer 3
Depth (m)	0–12	12–25	> 25
Dominant texture	Clay and silt loam (CL-ML)	Silty sand-clay and silt loam (SM, CL-ML)	Silty sand (SM)
SPT no	20–50	30 to > 50	> 50
UU cohesion (MPa)	0.015–0.03	0.015–0.025	0.02–0.04
CU cohesion (MPa)	0.01–0.02	0.01–0.02	0.015–0.025
UU internal friction	14–16	18–20	20–22
CU internal friction	20–24	26–28	30–32
Dry density (kg/m^3^)	1600–1700	1650–1750	1750–1850
Poisson’s ratio	0.38–0.4	0.33–0.36	0.3–0.32
Permiablity (m/s)	1E-7-1E-6	1E-7-1E-6	1E-8-1E-7
Compresibilty (MPa^−1^)	–	0.03–0.06	0.02–0.05
Vertical Young modulus (MPa)	20–30	35–50	50–70
Horizental Young modulus (MPa)	12–20	25–35	35–50
Reloading Young modulus (MPa)	40–60	70–100	100–140

The soil in this study is considered an elastic medium due to insignificant effects of plasticity in the final results of reflected and transmitted acoustic pressure. The acoustic wave behavior is examined by considering a relatively vast range of soil properties for each layer. Accordingly, besides the reference soil geotechnical properties, stiffer and softer versions are introduced in each layer, considering a proportional elevation and reduction of the density and module of elasticity, respectively. The mechanical properties of soil layers are presented in Table 2, including their reference, stiffer, and softer soil properties. The reference soils' mechanical properties (the first line associated with each soil layer in Table 2) are associated with the soil layers introduced previously in Table 1 and sourced from the results of borehole BH-23 excavated for the S03 station of Tabriz Urban Railway Line 2^38–40^. In addition, the stiffer and softer soil properties (associated with the second and third lines in Table 2, respectively) are assumed values used to study their effects on the acoustic wave behaviour when transmitted into the ground with a layered structure.

**Table 2:** Mechanical properties of soil layers.

Soil layer^a^	Young modulus (MPa)	Poisson's ratio	Density (g/cm^3^)	Depth (m)
Layer 1	50	0.4	1.7	0–12
	70	0.4	2.89	
	35	0.3	1.0	
Layer 2	85	0.36	1.75	12–25
	120	0.4	2.97	
	60	0.3	1.03	
Layer 3	120	0.3	1.80	> 25
	170	0.4	3.05	
	85	0.3	1.06	

^a^Each layer's first, second, and third rows represent their reference, stiffer, and softer soil properties.

The relative position of layers to the tunnel is investigated to comprehensively study the effects of varying soil properties and the tunnel-soil interaction. In addition to the 20-m buried depth mentioned earlier, two more tunnels with buried depths of 10 and 30 m (distances between the soil surface and the tunnel section center) are also considered in this investigation. Based on the soil layers' depths, shown in Table 2, for the cases of 20 and 30-m-buried depths, the tunnel is entirely located in the second and third layers, respectively. However, for the 10-m buried depth, most of the tunnel section is situated in the first layer, and a smaller portion is located in the second layer. As for the lining concrete, the three types of concrete introduced in Table 3 are applied.

**Table 3:** Mechanical properties of concrete and soil models.

Concrete type	Density (kg/m^3^)	Module of elasticity (GPa)	Poisson's ratio
Concrete I	2800	37	0.2
Concrete II	2700	35	0.2
Concrete III	2400	28	0.2
Soil	2200	8.23	0.3

### Validation of the proposed model

A reference model from Massah and Torabipour^32^ is considered to validate the proposed numerical model. This reference model contains an underground tunnel with a 5.3 m diameter and buried depth of 6.75 m (3.6 m soil cover) surrounded by a single-layer soil. The model dimensions were 40 m along the z-axis and 20 m along the y-axis, and it was simulated under an explosion of 900 kg TNT. The proposed model contains an underground tunnel with a 20-m buried depth, approximately three times (N = 3) deeper than the reference model. Based on the scaling law^41^, proper validation can be done if the proposed model uses the dimensions of 120 along the z-axis and 60 m along the y*-*axis. In addition, a 24,300 kg TNT explosion that is 27 times (N^3^) larger than the reference model's charge is applied in the simulation.

The investigated parameter to validate the proposed model is the transmitted acoustic pressure variation when changing the lining concrete type. Accordingly, three linear elastic concrete types, introduced by Massah and Torabipour^32^, are used. Table 3 shows the lining concrete types and the soil mechanical properties. The maximum transmitted acoustic wave pressure level for the reference and the proposed models are obtained and compared with each other. The transmitted acoustic wave pressure levels for both models are presented in Table 4.

**Table 4:** Transmitted acoustic pressure levels for the reference and the proposed models.

Concrete type	Transmitted acoustic pressure level (dB)	
	Reference model^32^	Proposed model
Concrete I	163.90	164.92
Concrete II	164.10	164.93
Concrete III	164.20	165.04

As observed in Table 4, for all the lining concrete types, both the reference and the proposed models yield approximately the same transmitted acoustic pressure level. The slight discrepancy between the results is due to the applied explosion mechanisms and equations.

## Results of the proposed model

To quantify acoustic wave behavior in an underground structure in a multilayer soil by changing various tunnel-related parameters, the reflected and transmitted acoustic pressure is determined in the air layer at the ground surface and the air inside the tunnel, respectively. The considered parameters include mechanical properties of soil in each layer, the buried depth, and the lining concrete type. In addition, the behavior of the acoustic wave is examined once an extra tunnel is added to the model in various locations with respect to the main tunnel.

### Effects of soil properties on acoustic wave behavior

This section presents the effects of soil mechanical properties on acoustic wave behavior. Mechanical properties associated with each soil layer are represented in Table 2. Soil properties in each layer are altered (based on their reference, stiffer and softer soil properties in Table 2) in all three buried depths to consider the tunnel-soil interaction properly. Several numerical analyses are applied for any specific buried depth; the first one is associated with the soil reference properties, and the subsequent ones are due to the soil characteristics alteration of one layer, while the others remain unchanged, and the model results are compared with that of the reference model (based on the reference soil properties in Table 2).

The reference soil's reflected and transmitted pressure values are 286.52 kPa, and 266.2 Pa for the 10-m buried depth, 306.94 kPa, and 178.39 Pa for the 20-m buried depth, and 306.99 kPa and 80.31 Pa for the 30-m buried depth. It should be noted that the reflected acoustic pressure values in this study consist of the reflected pressure values from the ground surface and the underground structure interior medium.

**Figure 4:**
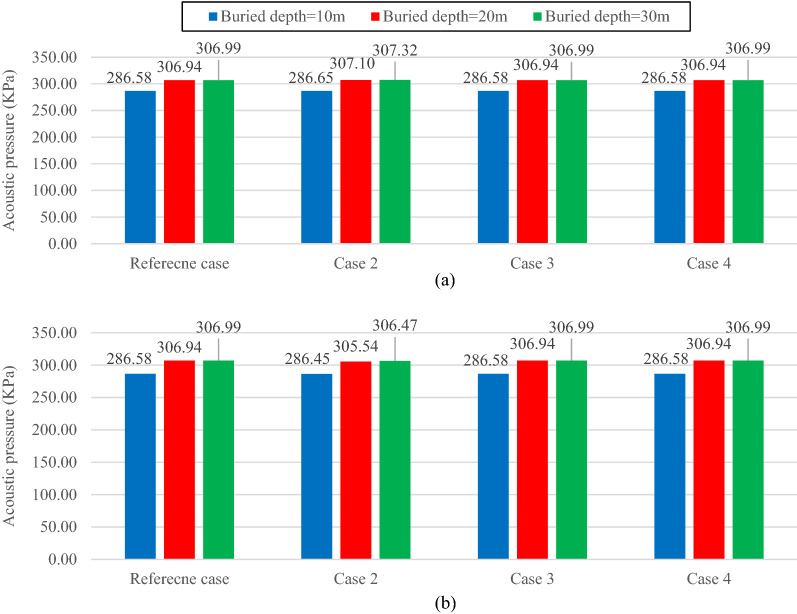
Maximum reflected acoustic wave pressure for the reference case model alongside the (**a**) stiffer and (**b**) softer soil cases.

**Figure 5:**
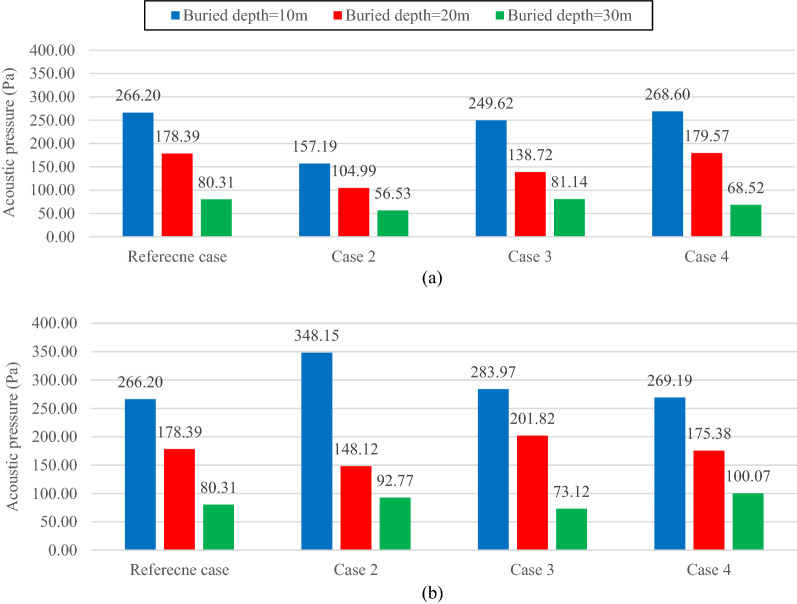
Maximum transmitted acoustic wave pressure for the reference case model alongside the (**a**) stiffer and (**b**) softer soil cases.

Concrete I (Table 3) is applied as the lining concrete in all models described in this section. Figure 4 shows the maximum reflected acoustic wave pressure with the reference soil characteristics alongside (a) the stiffened and (b) the softened ones for all the buried depths. Figure 5 presents the same for the transmitted acoustic wave. In both Figs. 4 and 5, cases 2, 3, and 4 are associated with altering the mechanical properties of soil in the first, second, and third layers, respectively.

Figure 4 shows that whether the soil stiffness increases (Fig. 4a) or reduces (Fig. 4b), regardless of the buried depth, the reflected acoustic waves are only affected by the properties of the first layer. The main reason behind this trend is the reflection coefficient effect between the soil and the air, which increases by stiffening the soil and reduces by softening it. An increase in the reflected acoustic wave value shows a more significant reflection coefficient. It is also observed that altering the properties of any layers except the first one hardly affects the reflected acoustic wave pressure. This shows the significant impact of the interaction of the first soil layer with air compared with the other factors (e.g., location of the underground structures, the interaction between the soil layers, and the interaction between the tunnel lining and the soil layers) on the reflected acoustic wave behavior. The same result was reported by Auersch^42^, in which, at higher frequencies (as in the case of an explosion), the top level of a layered soil significantly affects the foundation stiffness, and the acoustic wave reflected and transmitted by that foundation. In contrast, at lower frequencies, the deeper soil materials seem to have more influence on the foundation.

Figure 5 shows how the acoustic wave is transmitted when the properties of the soil layers are altered. The variation of the transmitted acoustic wave pressure depends on the relative position of the soil layers and the tunnel. Any changes in the properties of the soil layer containing the tunnel and the layers above them significantly affects the transmitted acoustic wave pressure. However, the stiffening or softening of the layers below the tunnel slightly affects the pressure value. Accordingly, in the case of a 10-m buried depth, stiffening the third layer (Fig. 5a) and softening it (Fig. 5b) cause the transmitted acoustic pressure to vary by 0.9% and 1.12%. The same scenario for a buried depth of 20 m leads to minimal variations of 0.66% and 1.69% of the transmitted acoustic wave pressure for the stiffer and softer third layer soil.

On the other hand, considering the case of a 10-m buried depth shows that if even a small section of the tunnel is located in a soil layer, that layer can significantly affect the transmitted acoustic wave behavior. In such a model, the transmitted acoustic pressure is reduced by 40.95% and 6.23% (compared with the reference case), respectively, with the first and the second stiffer layers (Fig. 5a). The transmitted wave pressure increases by 30.78% and 6.67% (compared with the reference case), respectively, with the first and the second softer layers (Fig. 5b). The same behavior pattern is observed in the cases of 20 and 30-m buried depths, where stiffening the second and third layers reduces the transmitted wave pressure by 22.23% and 14.6%, respectively. In contrast, decreasing the soil stiffness in such layers results in a 13.13% and 24.6% increase in acoustic pressure, respectively.

When increasing a soil layer stiffness, the soil particle and particularly the lining vibrations (accelerations) are reduced. This reduction in acceleration leads to the mitigation of the acoustic wave energy propagated through the soil and transmitted inside the tunnel. Therefore, amplifying the soil layer stiffness, which contains the tunnel (partial or complete), diminishes the transmitted acoustic wave energy and hence the acoustic wave pressure transmitted into the tunnel.

As for the layers above the tunnel level, alongside the soil stiffness effect, the reflection coefficient between the soil layers is another determinant factor affecting the transmitted acoustic wave behavior. Considering the case of 20-m buried depth, for both the stiffer (Fig. 5a) and the softer (Fig. 5b) first layer, reduction of 41.14% and 16.97% are respectively experienced by the transmitted acoustic wave pressure (compared with the reference cases). In both instances, the calculated reflection coefficients between the first and the second layers appear to be greater than the ones of the reference model. It explains the transmitted wave pressure drops in both cases. However, the decreased values depend on how the soil stiffness varies for each case. The soil particle vibration and the acoustic wave propagated through the layer are reduced for the case with stiffer first layers. Therefore, higher pressure drops are observed in comparison with the softer case.

On the other hand, in the case of 30-m buried depth, no such trend occurs when altering the first layer. In this case, despite the reflection coefficient rise between the first and the second layers, the transmitted acoustic wave behavior is highly influenced by the soil stiffness of the first layer. When this layer is stiffer, the pressure drops (Fig. 5a), and when it is softer, the pressure increases (Fig. 5b). Such independence of the results from the reflection coefficient in the aforementioned case is due to the attenuation of this factor caused by the considerable distance between the tunnel and the interface of the first and the second layers. By reducing the distance, the reflection coefficient effect on the transmitted acoustic wave behavior is amplified, as shown in cases of 20 and 30-m buried depths.

The reflection coefficient mentioned earlier is defined based on the acoustic impedance of the two adjacent mediums through which the acoustic wave is transmitted. This coefficient is the ratio of reflected wave intensity from the interface of two adjacent mediums to the transmitted wave intensity passed from that interface. In this study, the reflection coefficient is calculated as the squared ratio of the difference between two adjacent mediums' acoustic impedance and the sum of the acoustic impedances. Aristizábal-Tique et al.^34^ used the same definition, except its second power. Comparing this definition with Guan et al.'s^43^ evaluation of the sound absorption of the porous media indicates the same concept imagined, except that the present study uses the characteristic acoustic impedance in calculating the reflection coefficient applied in nondispersive linear acoustics in one dimension. This coefficient shows "the amount of acoustical energy being reflected when propagating waves meet an obstacle or a different medium of propagation"^43^. It indicates how much radiated acoustic wave energy reflects from the two mediums' interface on a scale of zero to one, where zero means that all the acoustic wave passes through the interface and one indicates the complete reflection of the radiated wave. The reflection coefficient between the air and the surface soil layer, between the various soil layers, and between the soil layers and the tunnel lining affects the transmitted and reflected acoustic wave pressure, but it is not the only factor. The other contributing factors are amplifying effect of soil particle vibration and the time when the acoustic wave impacts the interface.

### Effect of tunnel buried depth on acoustic wave behavior

**Figure 6:**
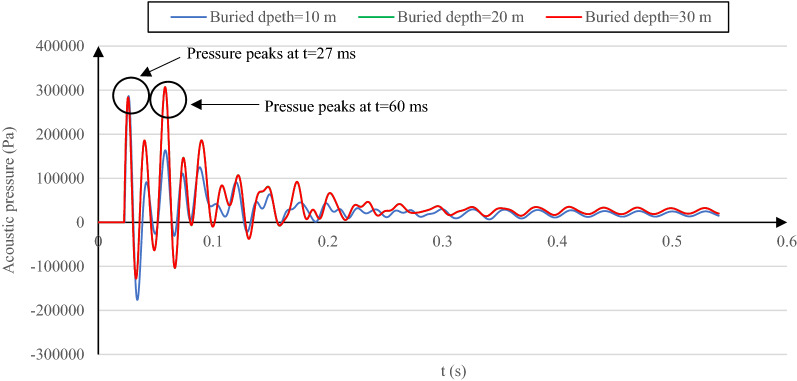
Reflected acoustic wave pressure–time history for the cases of 10-m, 20-m, and 30-m buried depth with reference soil properties. Note that the data associated with 20 and 30-m depths are almost coincident.

Buried depth is another investigated parameter to quantify its effect on the acoustic wave in a layered soil medium. The reference properties of soil layers are used in this section. The maximum pressure value increases with the buried depth increase for the reflected acoustic wave, as illustrated in Fig. 4. However, Fig. 4 does not include the time shift of the absolute maximum pressure alongside its value when changing the buried depth. The reflected acoustic wave pressure–time history is represented in Fig. 6 for the three buried depths to show both variations.

As observed in Fig. 6, the maximum reflected acoustic pressure increases with the buried depth growth; the peak time is shifted from t = 27 ms in the case of a 10-m buried depth to (approximately) t = 60 ms for the two other cases. Such value and time shifts of the reflected acoustic wave are due to how the acoustic wave energy and the soil particle vibration vary with time. Accordingly, the soil particle acceleration is the first parameter influenced by the acoustic wave energy level and the resultant particle vibration. Consequently, the total acceleration value at the soil surface level can represent the acoustic wave energy propagated through the soil and reflected. So, interpreting the soil total acceleration behavior when changing buried depth allows explaining such behavior seen in Fig. 6.

**Figure 7:**
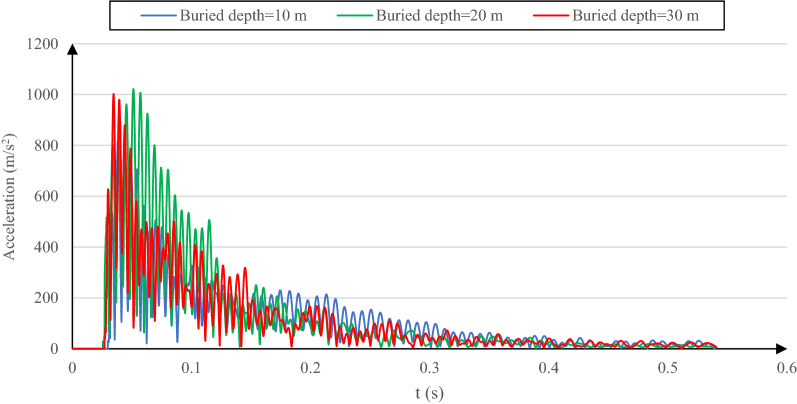
Total surface soil acceleration variation versus time for the cases of 10-m, 20-m and, 30-m buried depth with reference soil properties.

The total acceleration time history of the surface soil for various buried depths is shown in Fig. 7. Due to the soil structure interaction and particularly the first layer's constant properties when changing the buried depth, the air–soil interaction and the reflection coefficient factors are not applicable when analyzing such behavior.

The surface soil acceleration represented in Fig. 7 is generated by the free air explosion accompanied by the acoustic wave reflected with a time lag. These two acoustic wave sources were identified and investigated exclusively by Albert et al.^44^. The way these two soil acceleration sources interact is the fundamental reason behind how the reflected acoustic wave behaves when changing the buried depth in the numerical model. Taking the case of 30-m depth (with the highest reflected acoustic pressure value) as an example, the surface acceleration peak is obtained before the reflected pressure absolute peak (Fig. 6). While the air explosion is still in progress (Fig. 7), it induces the primitive air explosion-induced acoustic wave amplification by the acoustic wave reflected from the tunnel and the soil layers interfaces. As a result of such acoustic wave strengthening, the reflected acoustic wave pressure absolute peak increases compared to the local one (at t = 27 ms), and its instant is modified to t = 60 ms. However, in the case of a 20-m buried depth, although a relatively higher surface soil acceleration than the 30-m buried depth case, a lower reflected acoustic wave pressure is observed due to the lower acoustic wave amplification at the surface level. This is justified because the surface soil acceleration peak instant occurs after the air explosion. The same reason added to the lowest surface soil acceleration magnitude among all cases stops the acoustic wave amplification at the surface level in the case of 10-m buried depth and induces the lowest reflected acoustic wave value with no peak instant shift. The fundamental reason for such behavior (10-m buried depth) is because, for this specific lining concrete, the energy radiated into the soil pile is sent out by the tunnel lining before reaching the soil layer interface. Thus the energy amplification does not occur. A similar conclusion was made by Aristizábal-Tique et al.^34^ about the peak time shift of the reflected acoustic wave associated with the three buried depths. It happened due to the phase change and the time lag between the reflected wave from the interior space of soil (the buried structure and the soil layers interface) and the explosion. The position of the acoustic wave pressure peaks is shifted due to the density difference between the soil and the buried structure and is sensitive to the depth of the underground structure and the wave propagation in the first layer of the soil.

**Figure 8:**
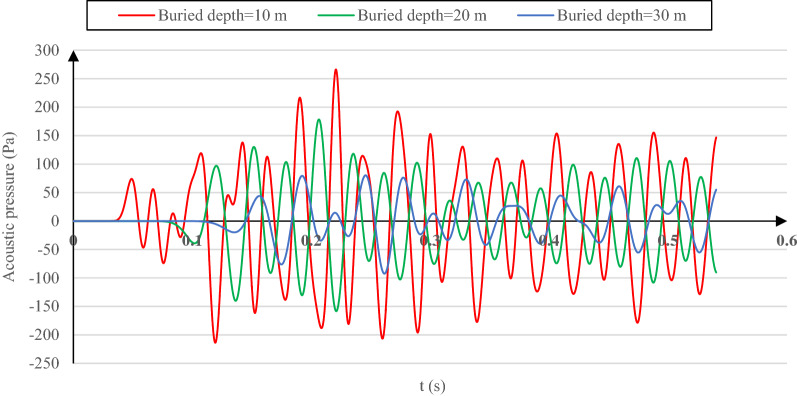
Transmitted acoustic wave pressure time histories for the cases of 10-m, 20-m and, 30-m buried depth with reference soil properties.

The maximum pressure value of transmitted acoustic waves (Fig. 5) and their time history for all buried depths (Fig. 8) indicate that increasing the buried depth results in transmitted acoustic wave pressure drop in the same soil structure. This trend is expected due to the higher acoustic wave energy deterioration in reaching the tunnel with greater buried depths.

### Effect of lining concrete on acoustic wave behavior

**Figure 9:**
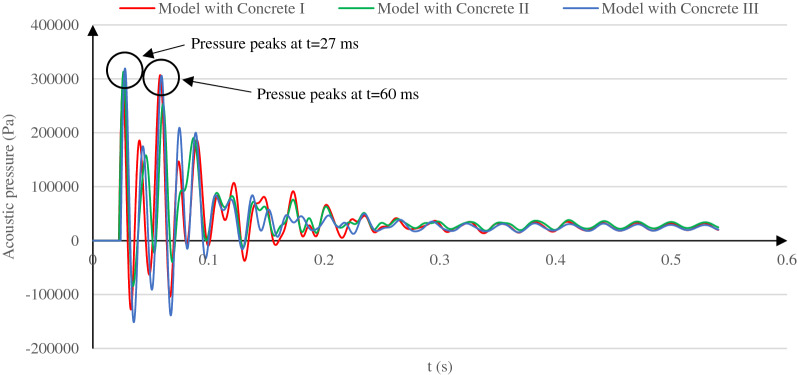
Pressure time histories of the reflected acoustic wave for the models with Concrete I, Concrete II, and Concrete III as the tunnel lining concrete.

This section discusses the lining concrete impact on the behavior of the acoustic wave for the buried depth of 20 m, the reference soil properties, and two concrete types. Time histories of the reflected acoustic wave pressure and the corresponding maximum value of such pressure associated with each concrete type are illustrated in Fig. 9.

Based on the pressure time histories in Fig. 9, when the lining concrete stiffness reduces, the absolute peak value of the reflected acoustic wave rises. A time shift in the occurrence of such absolute value is observed from t = 60 ms in the concrete I model (with an absolute pressure of 306.94 kPa) to t = 27 ms in the two other softer lining cases (with absolute pressures of 313.33 kPa and 319.29 kPa in Concrete II and III models, respectively). These behaviors are due to the lining concrete time-dependent effect on the reflected acoustic waves. Shortly after the explosion, overpressure peaks at t = 21.88 ms (Fig. 1), and the explosion's acoustic pressure mainly dominates the measured acoustic wave at the surface. It is less affected by the acoustic waves reflected from the soil and tunnel interior space. In this situation, reducing the lining concrete stiffness and, consequently, the far end boundaries damping reduction leads to the lining energy attenuation reduction. This phenomenon results in a reflected acoustic pressure growth at the beginning of the analysis, which is reflected by an increased pressure value at t = 27 ms when lowering the lining concrete stiffness, Fig. 9.

As time passes, high-amplitude acoustic waves are reflected from the tunnel lining and the soil layers’ interfaces, especially at the explosion process end. Consequently, changing the concrete stiffness affects the acoustic waves' energy variation reflected back to the surface and thus their pressure value. Accordingly, reducing the lining concrete stiffness reduces the reflection coefficient between the lining and the soil. It then reduces the reflected waves from the lining, but on the other hand, it causes a lining particle's vibration growth, leading to the reflected acoustic wave amplification. These inhomogeneous outcomes result in a non-uniform reflected acoustic wave pressure variation at the explosion period end. This non-uniform variation is observed in the reflected pressure value at t = 60 ms. It drastically diminishes in the model with Concrete II and then increases back to the primary value in the third model.

**Figure 10:**
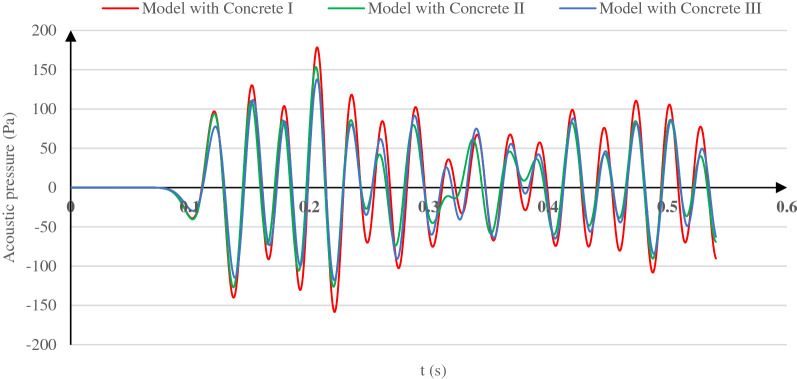
Pressure time histories of the transmitted acoustic wave for the models with Concrete I, Concrete II, and Concrete III as the tunnel lining concrete.

Vanishing the acoustic wave energy before reaching the tunnel lining for different lining concrete types also impacts the transmitted acoustic wave pressure. Figure 10 presents the transmitted acoustic wave pressure–time history and the maximum values of such pressure for each lining concrete type. As observed in Fig. 10, the transmitted acoustic pressure decreases from 178.39 Pa in the concrete I model by 14.01% and 22.88% for the concrete II and concrete III models by reducing the lining concrete stiffness. This behavior is due to the acoustic wave energy decreasing from the soil surface to the lining and how the acoustic wave energy varies in the tunnel vicinity.

As discussed earlier, the total acceleration is a better parameter to represent the acoustic wave energy level of soil and lining concrete. Therefore, to illustrate the acoustic wave energy variation for different concrete models, the maximum total accelerations for soil in various elevations in the vicinity of the lining and the maximum acceleration for lining with the three concrete types are represented in Table 5. The elevations, which are considered based on the tunnel section center, are the highest lining section point (Elev. 14.85 m), the middle of the lining section (Elev. 10 m), and the lowest lining section point (Elev. 5.15 m).

**Table 5:** Lining and soil maximum total acceleration for the three concrete models.

Concrete types	Lining maximum acceleration (m/s^2^)	Soil maximum acceleration (m/s^2^)		
		At elev. 14.85 m	At elev. 10 m	At elev. 5.15 m
Concrete I	23.96	35.94	35.94	26.98
Concrete II	22.56	35.43	33.65	28.68
Concrete III	18.96	31.82	31.82	23.56

Table 5 shows that the highest soil acceleration around the tunnel and the highest lining acceleration are in the Concrete I and Concrete II models. Consequently, the lining and soil acceleration variation pattern in the tunnel lining vicinity and how the transmitted acoustic wave pressure varies when lowering the concrete stiffness (Fig. 10) indicate the lining concrete effects on the acoustic energy and how the transmitted acoustic wave pressure varies.

### Extra underground structure

**Figure 11:**
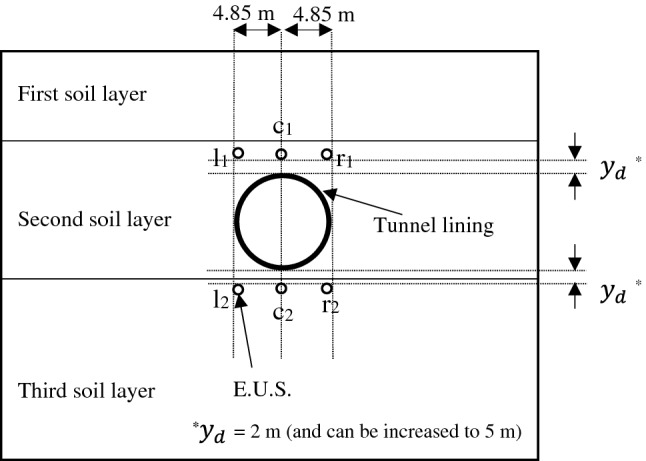
Schematic of various positions of E.U.S. relative to the main tunnel.

In a buried structure system, underground utility lines and their maintenance and reparations can interfere with the construction and operation of the main structure. Locating the exact positions of these lines by applying the acoustic wave necessitates an investigation of the reflected and transmitted acoustic wave behavior for wide structure positions for the main tunnel. An extra underground structure (E.U.S.) is set up in various relative positions to the main tunnel with the 20-m buried depth (reference case) to study the behavior of acoustic waves (Fig. 11). The inner diameter of the E.U.S. is 0.8 m, surrounded by a 0.1-m-thick lining made of Concrete I.

**Figure 12:**
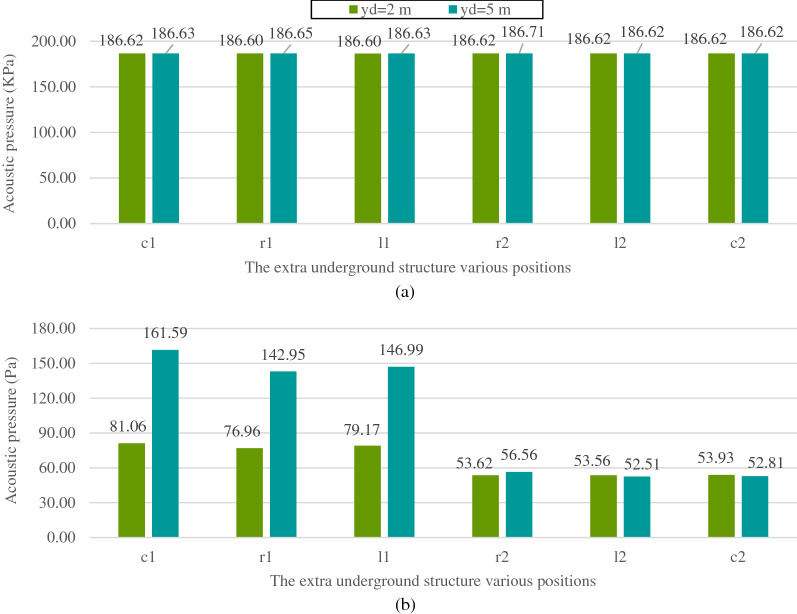
Maximum (**a**) reflected and (**b**) transmitted acoustic wave pressure for various positions of the extra underground structure.

The E.U.S.s aligned with the vertical centerline of the cross-section of the main tunnel is named by the letter "c". The E.U.S.s on the right and left sides of the main tunnel are denoted by "r" and "l", respectively. The subscripts "1" and "2" indicate whether the E.U.S. is above or below the main tunnel, respectively. In addition, an extra variable ${y}_{d}$ with two values of 2 m and 5 m is introduced as the vertical distance between the main tunnel and the E.U.S. to allow investigation of how the acoustic waves react when altering the soil layer containing the E.U.S.. Figure 12 shows the maximum reflected and transmitted (from the main tunnel) acoustic wave pressures for various E.U.S. positions with the two values of ${y}_{d}$ are illustrated.

By comparing the results represented in Fig. 12 with the reflected and transmitted acoustic wave pressures of the same model without E.U.S. (306.94 kPa and 178.39 Pa, respectively), it is found that adding the E.U.S. to the model acts as an energy absorber mechanism. It makes the radiated acoustic wave energy lead out of the system and causes a reduction in the reflected and transmitted acoustic wave pressures. Besides the energy absorption effect, the E.U.S. reflects the radiated acoustic waves toward the main tunnel and the soil surface. As shown in Fig. 12b, for both ${y}_{d}$ values the transmitted acoustic wave pressure drops for the E.U.S.s below the tunnel compared with those above it. The reflected acoustic waves radiated from the E.U.S. towards the soil surface, and the main tunnel decreases when the E.U.S. is located below the tunnel. The same behavior is observed for the reflected acoustic waves (Fig. 12a), especially in the case of ${y}_{d}$=5 m. Increasing the ${y}_{d}$ value reduces the acoustic pressure (reflected and transmitted) when the E.U.S. elevation decreases. Increasing the ${y}_{d}$ value has a more significant impact on the E.U.S.s above the main tunnel than those below it.

The E.U.S.s above the main tunnel move from the second to the first layer by increasing the ${y}_{d}$, but the E.U.S.s below the main tunnel remain in the same soil layer (the third layer) for both values of ${y}_{d}$. Moving the E.U.S. from the second to the first layer causes a reflection coefficient increase between the E.U.S. and the soil. Therefore, the reflected acoustic wave radiated from the E.U.S. towards the main tunnel, and the ground surface increased. Consequently, the acoustic pressures reflected back to the surface and transmitted into the main tunnel increase, as shown in Fig. 12. If the E.U.S. is located above the soil layer interface, it prevents attenuation of the reflected acoustic wave (from the E.U.S.) through that interface. For the E.U.S.s below the main tunnel, such layer alteration does not occur, and the acoustic wave pressure changes in a more limited range by increasing ${y}_{d}$.

The importance of investigating the acoustic wave behavior emitted in an underground medium was pointed out earlier in the introduction to acoustic wave-based methods. The propagated signals from a buried structure fracture are severely influenced by the structural components and the soil surrounding it. Using the acoustic emission (A.E.) method as an underground structure health monitoring technique, the lack of knowledge about the acoustic wave behavior when interacting with these parameters would result in misinterpretation of the recorded signals. For example, the presence of an extra underground structure (like the utility lines) in the vicinity of the main tunnel can interfere with the signals emitted from the tunnel lining to the surface and cause misinterpretation of the structural health of the main tunnel. Therefore, investigating the acoustic wave behavior in the presence of E.U.S. (in various positions with respect to the main tunnel) in comparison with the original scenario (main tunnel without E.U.S.) helps avoid misinterpreting the recorded signals.

The soil structure (single layer or multilayer), the relative position of the tunnel and the soil layers, and the resultant multi-peak reflected acoustic wave pressure (discussed in “Effect of tunnel buried depth on acoustic wave behavior” section) can also affect the interpretation of the reflected acoustic signals in the A.E. method. This may lead to incorrect judgment of a probable fracture on tunnel lining if not appropriately investigated. The same applies to the lining concrete stiffness and the soil mechanical properties, which drastically influence the acoustic wave behavior and the consequent interpretation of the signals.

## Conclusions

Knowing how the acoustic waves behave when interacting with an underground structure provides essential and valuable information for comprehensive understanding and precise interpretation of the recorded signals associated with the acoustic wave-based method, especially when dealing with multilayer soil structures. This research studied the behavior of the acoustic wave when propagating inside an underground structure media with different soil properties, buried depths, and lining concrete types by considering a multilayer soil structure under a free air explosion. An extra underground structure was then introduced to the model to evaluate its impact on acoustic wave behavior. A 3D model of an underground tunnel, including the tunnel lining, a three-layer soil surrounding the tunnel, and the air inside the tunnel and at the surface, was developed using the commercial finite element code, ABAQUS. A free air explosion was used as the acoustic wave source, and the reflected and transmitted acoustic wave pressures were measured. With regards to the maximum acoustic wave pressure values and how they behave with time, the following points are noted:

- The mechanical characteristics of the soil layer directly affect the transmitted acoustic waves depending on the relative position of the layer in regard to the tunnel. The reflected acoustic wave is only affected by modifying the properties of the first layer. The most significant effect was observed for the soil layer containing the tunnel. However, the alteration of soil properties in a soil layer below the mentioned layer leads to an insignificant variation in transmitted acoustic wave pressure.
- The transmitted acoustic wave decreases when the tunnel is buried in deeper soil layers due to the higher attenuation of an acoustic wave propagating towards the tunnel. On the other hand, the reflected acoustic wave pressure increases with absolute peak instant shift, originating from the amplification of the acoustic wave at the soil's surface by the one reflected back from the interior space of the soil and subsurface tunnel.
- In the case of using different lining concrete types, the transmitted acoustic wave pressure depends on the variation of the acoustic wave energy in the vicinity of the tunnel lining. The lowest acoustic wave energy is observed around the tunnel with the lowest lining concrete stiffness and the lowest transmitted acoustic pressure. In contrast, higher stiffness concrete lining causes lower attenuation of the acoustic wave energy. The lining concrete stiffness has a time-dependent effect on the reflected acoustic waves. By reducing the stiffness of the concrete, the lining energy attenuation reduces shortly after the explosion overpressure peaks, and the reflected acoustic pressure increases. At the explosion process end, the lower stiffness of concrete reduces the reflected waves from the lining but leads to amplification of the reflected acoustic wave.
- Adding an extra underground structure to the model decreases the reflected and transmitted acoustic waves. Increasing the vertical distance between the extra structure and the main tunnel increases the variation amplitude of the acoustic waves. In addition, if the additional structure is located in the same soil layer as the main tunnel, the transmitted pressure deviation from a case without the extra structure increases, which can be interpreted as the interference growth of the acoustic signals emitted from the main tunnel. This point can help in a more precise interpretation of the recorded signals in the methods associated with locating the subsurface structures and their health monitoring.
- One of the key features affecting the acoustic wave behavior and distinguishing between a single layer and a multilayer soil structure is the acoustic wave reflection from the soil layers' interfaces. This is a dominant factor when the tunnel buried depth is altered. It also causes two peaks with relative values for the lining concrete type and the tunnel buried depth in the reflected acoustic wave time history. This phenomenon has not been reported in previous studies nor observed for a single-layer soil structure. It can be falsely interpreted as possible damage by signal processing of the acoustic wave-based method of a multilayer soil structure.
- The studies in acoustic structural health monitoring can be expanded by introducing a fracture on the tunnel's lining and investigating the reflected and transmitted acoustic waves in the presence of that damage. Also, the realistic modeling of the lining, including its separate segments, can lead to more accurate results. The results of the present study can be used in future studies to provide a more realistic and more precise interpretation of the recorded signals for structural health monitoring and locating underground structures, especially in a media with multilayer soil structures.

## Data Availability

The data supporting this study's findings are available from the corresponding author, R. K., upon reasonable request.

## Code availability

ABAQUS was utilized for simulation.
